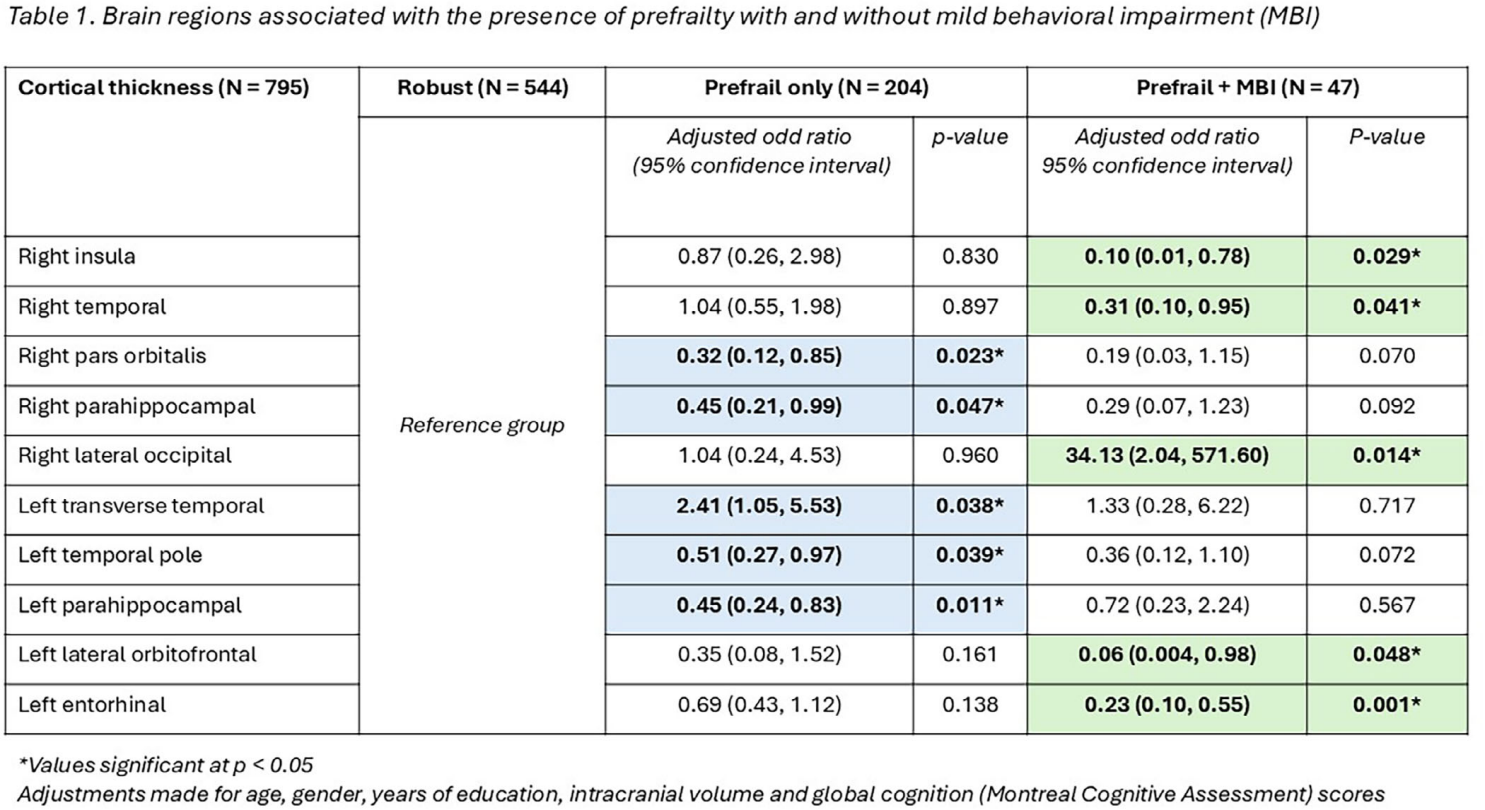# Behavioural Frailty: A Novel Exploration of the Association Between Co‐Occurring Prefrailty and Mild Behavioural Impairment with Cortical Thickness

**DOI:** 10.1002/alz70857_106690

**Published:** 2025-12-26

**Authors:** Kiirtaara Aravindhan, Ashwati Vipin, Yi Jin Leow, Nagaendran Kandiah

**Affiliations:** ^1^ Lee Kong Chian School of Medicine, Nanyang Technological University, Singapore, Singapore; ^2^ nil, nil, nil, Nicaragua; ^3^ Neuroscience and Mental Health Programme, Lee Kong Chian School of Medicine, Nanyang Technological University, Singapore, Singapore; ^4^ National Healthcare Group, Singapore, Singapore

## Abstract

**Background:**

Research reveals an association between frailty and mild behavioural impairment (MBI), both of which have been individually associated with cognitive impairment leading to dementia. However, their combined effect remains understudied. This study explores a potential “behavioural frailty,” the co‐occurrence of prefrailty and MBI, and its association with cortical thickness in a Southeast Asian cohort.

**Method:**

795 community‐dwelling participants were recruited from the Biomarker and Cognition Study Singapore, all of whom had completed the 34‐item Mild Behavioural Impairment Checklist, Fried Frailty Phenotype (FFP) questionnaire, neuropsychological assessment, and had undergone a T1‐weighted 3T magnetic resonance imaging (MRI). Participants were classified as MBI‐positive based on established cut‐off of ≥5.5, and prefrail based on presence of ≥1 criteria on the FFP. Structural MRI was acquired from T1‐weighted MPRAGE sequence and images pre‐processed using FreeSurfer version 7.2. Observation of participant characteristics, group differences and regression were performed using SPSS, with statistical significance set a *p* <0.05.

**Result:**

Mean age of participants were 59.44±9.10 with 61.9% female. 25.7% were prefrail, while 5.9% had prefrailty with MBI. Significant differences in white matter hyperintensity volume and neurofilament‐light mean concentration was observed between the groups. The right hemisphere presented significant differences in cortical thickness were observed in the insula, transverse temporal, temporal, supramarginal, superior temporal, pars orbitalis, parahippocampal and entorhinal regions. While the left hemisphere presented significant differences in the cortical thickness of the insula, temporal pole, parahippocampal and entorhinal regions. Prefrailty without MBI were significantly associated with reduced cortical thickness in the right pars orbitalis, right parahippocampal and left parahippocampal. While significant associations were observed between decreased cortical thickness in the right insula, right temporal, left lateral orbitofrontal and left entorhinal with the co‐occurrence of prefrailty and MBI. All associations were adjusted for age, gender, education years, intracranial volume, and global cognition scores.

**Conclusion:**

Findings provide an overview of structural correlates associated to presence of prefrailty with and without MBI. “Behavioral frailty” was associated with cortical thinning in the right insula, right temporal lobe, left lateral orbitofrontal cortex, and left entorhinal cortex, which are engaged in various cognitive and emotional functions in the brain.